# Association of *NCF2*, *NCF4*, and *CYBA* Gene Polymorphisms with Rheumatoid Arthritis in a Chinese Population

**DOI:** 10.1155/2020/8528976

**Published:** 2020-10-20

**Authors:** Tian-Ping Zhang, Rui Li, Qian Huang, Han-Feng Pan, Dong-Qing Ye, Xiao-Mei Li

**Affiliations:** ^1^The First Affiliated Hospital of University of Science and Technology of China, Hefei, 230001 Anhui, China; ^2^Department of Nosocomial Infection Management, The First Affiliated Hospital of Anhui Medical University, Hefei, 230022 Anhui, China; ^3^School of Public Health, Anhui Medical University, Anhui Provincial Laboratory of Inflammatory and Immune Diseases, Hefei, 230032 Anhui, China

## Abstract

**Objective:**

Recent studies have focused on the special roles of NADPH-oxidase in multiple autoimmune diseases. Nevertheless, the association of genetic variation in NADPH-oxidase genes with rheumatoid arthritis (RA) was not extensively studied in a Chinese population. We performed this study to examine the association of *NCF2*, *NCF4*, and *CYBA* gene polymorphisms with RA susceptibility in a Chinese population.

**Methods:**

Six single nucleotide polymorphisms (SNPs) (*NCF2* rs10911363, *NCF4* rs1883112, rs4821544, rs729749, *CYBA* rs3794624, and rs4673) were genotyped in a cohort composed of 593 RA patients and 596 normal controls. Improved multiple ligase detection reaction (iMLDR) was used for genotyping.

**Results:**

We observed that *NCF4* rs4821544 CT genotype and C allele frequencies in RA patients were significantly decreased when compared to controls (CT vs. TT: *P* = 0.043; C vs. T: *P* = 0.031), and rs4821544 polymorphism was significantly associated with an increased RA risk under the dominant model (TT vs. CT+CC: *P* = 0.031). Our results also indicated that rs729749 CT genotype frequency was significantly lower in RA patients than that in controls (CT vs. CC: *P* = 0.033). Moreover, the rs729749 CT genotype frequency was also significantly decreased in RA patients in males (CT vs. CC: *P* = 0.024). No significant association between *NCF2* and *CYBA* gene polymorphisms and RA susceptibility was observed. There were significant associations between rs4821544 TT genotype and T allele frequencies and anti-CCP in male RA patients.

**Conclusions:**

In summary, *NCF4* rs4821544 and rs729749 polymorphisms might contribute to RA susceptibility, while *NCF2* and *CYBA* gene polymorphisms were not associated with RA susceptibility.

## 1. Introduction

Rheumatoid arthritis (RA) is a systemic, chronic autoimmune disease affecting approximately 1-2% of the population worldwide [1]. The disease is characterized by immunologic inadequacy and chronic inflammation and predominantly causes deformity and destruction of the joints [2]. The pathogenesis of RA is not completely identified and generally considered to be related to the interaction between environmental factors and genetic predisposition [3]. Despite many efforts devoted to the studies of predominant genetic markers, only few genetic loci including human leukocyte antigen (*HLA*) locus and protein tyrosine phosphatase nonreceptor type 22 (*PTPN22*) have been identified to be associated with RA [4, 5]. Therefore, it is necessary to continue to analyze whether immune-modulating gene variations are associated with RA.

Recent studies indicated that elevated levels of reactive oxygen species (ROS) and reactive nitrogen species (RNS) were found in several autoimmune diseases such as RA and systemic lupus erythematosus (SLE) and involved in inflammatory processes [6, 7]. The production of ROS was a physiological defense against microbial infection, and ROS had an important antibacterial effect on a variety of pathogens. However, the aberrant generation of ROS in autoimmune inflammation could result in tissue damage [6]. Moreover, ROS also had important regulatory functions in the immune system [8]. The NADPH-oxidase (NOX) complex, which was critical for ROS generation, was composed of gp91phox, p22phox, IM7phox, p67phox, and p40phox encoded by *CYBB*, *CYBA*, *NCFl*, *NCF2*, and *NCF4* genes, respectively [9, 10]. The ability to induce ROS extends to phagocytes and dendritic cells, also implying the key functions of the NOX complex in the immune system [11].

The hypothesis that genetically controlled reduced production of ROS caused by *NOX* gene variations could affect the risk of autoimmune diseases and ROS-regulated chronic autoimmune inflammatory diseases was supported by several studies [12, 13]. Olsson et al. suggested that *NCF1-339* T allele frequency in SLE patients was increased in comparison to controls. In addition, the *NCF1-339* T allele reduced extracellular ROS production in neutrophils and resulted in the elevated expression of type 1 interferon-regulated gene [12]. It was remarkable that there were several researches exploring the association between *NOX* gene polymorphisms and RA susceptibility [13, 14]. Zhao et al. demonstrated that the p.Arg90His variant in *NCF1*, which was observed to cause reduced ROS production, predisposed to RA in a Korean population [13].

Apart from *NCF1*, several single nucleotide polymorphisms (SNPs) in *NCF2*, *NCF4*, and *CYBA* genes were reported to be associated with autoimmune diseases [15–17]. The results by Olsson et al. indicated that the *NCF4* rs729749 variant was involved in the development of RA in a Swedish cohort [16]. However, the association between *NCF2*, *NCF4*, and *CYBA* genetic variants and RA risk in a Chinese population had not been reported. To investigate whether *NCF2*, *NCF4*, and *CYBA* gene polymorphisms are associated with RA susceptibility, we performed this case-control study in a Chinese population.

## 2. Materials and Methods

### 2.1. Study Populations

In this study, a total of 593 RA patients including 101 males and 492 females were recruited from Anhui Provincial Laboratory of Inflammatory and Immune Diseases. All patients were diagnosed depending on the 1987 American College of Rheumatology revised criteria [18]. Then, a normal cohort of 97 males and 499 females, which had no history of inflammatory/autoimmune diseases and cancer, was enrolled from the same region. The average ages of RA patients and normal controls were 51.59 ± 6.68 years and 52.32 ± 12.63 years, respectively, and there was no significant difference in age distribution between RA patients and controls. The demographic and clinical data of RA patients were collected from the medical records and reviewed by a rheumatologist, and the clinical data mainly included anticyclic citrullinated peptide (anti-CCP) and rheumatoid factor (RF). All RA patients and normal controls were enrolled after their written informed consent had been obtained, and the study protocol was approved by the Medical Ethics Committee of Anhui Medical University.

### 2.2. SNP Selection

Several previous studies had shown that *NCF2* gene polymorphisms (rs17849502, rs35937854, rs13306575, rs789181, and rs10911363) were significantly associated with the development of autoimmune diseases [16, 19, 20]. However, only the minor allele frequency (MAF) of rs10911363 was greater than 0.05 in the CHB population. Hence, rs10911363 in *NCF2* was included for genotyping in the present study. Because of limited research on the *NCF4* and *CYBA* genetic variants in RA, we utilized genotype data of the CHB from Ensembl genome browser 85 and CHBS_1000g and selected the tagSNPs capturing all the common SNPs located in the chromosome locus transcribed into *NCF4* and *CYBA* and their flanking 2000 bp region by the Haploview 4.0 software (Cambridge, MA, USA). We finally selected rs1883112, rs4821544, and rs729749 in *NCF4* and rs3794624 and rs4673 in *CYBA* for genotyping, and the *NCF4* rs4821544, rs729749, *CYBA* rs3794624, and rs4673 had also been studied in the Swedish population [16]. Above SNPs accorded with MAF ≥ 0.05 in CHB and *r*^2^ threshold > 0.8.

### 2.3. DNA Extraction and Genotyping

A total of 5 ml peripheral blood sample was collected from all study populations by tubes containing ethylenediaminetetraacetic acid (EDTA). Then, genomic DNA was prepared from the peripheral blood leukocytes according to the standard procedures with the FlexiGene DNA Kit (Qiagen, Valencia, CA).

The genotyping was conducted using improved multiple ligase detection reaction (iMLDR) genotyping assays, with the technical support of Genesky Biotechnologies Inc., Shanghai. Those subjects with 100% genotype success for all SNPs were involved in the final analysis.

### 2.4. Statistical Analysis

Statistical analysis was done in the SPSS 23.00 (SPSS Inc., IL, USA). The chi-square (*χ*^2^) test was used to analyze the association of the genotype and allele frequencies of above SNPs and RA patients. Odds ratios (OR) and 95% confidence interval (CI) were also evaluated using logistic regression analyses. Two genetic models including the dominant model and the recessive model were also analyzed. The Hardy-Weinberg equilibrium (HWE) test was conducted in the control group. Haplotype was assessed using the SHEsis software (http://analysis.bio-x.cn/myAnalysis.php) [21]. A two-sided *P* < 0.05 was considered as statistically significant.

## 3. Results

### 3.1. Association of *NCF2*, *NCF4*, and *CYBA* Gene Polymorphisms with RA Susceptibility

In normal controls, the genotype frequencies of *NCF2* rs10911363, *NCF4* rs1883112, rs4821544, rs729749 and *CYBA* rs3794624, and rs4673 were all in compliance with the HWE (all *P* > 0.05). The allele and genotype frequencies of these SNPs are shown in Table 1. There was no significant difference in the genotype frequencies of *NCF2* rs10911363 between RA patients and controls. Similarly, the allele frequencies of rs10911363 in RA patients were comparable to controls. Then, we stratified all subjects by sex and analyzed the association between rs10911363 and RA susceptibility in males and females, respectively. No significant association was found.

Regarding the genotype and allele frequencies of *NCF4* rs1883112, rs4821544, and rs729749, we noted that the CT genotype and C allele frequencies of rs4821544 in RA patients were significantly decreased when compared to controls (CT vs. TT: OR = 0.752, 95% CI: 0.571-0.991, *P* = 0.043; C vs. T: OR = 1.316, 95% CI: 1.026-1.688, *P* = 0.031). Moreover, the rs4821544 polymorphism was significantly associated with an increased RA risk under the dominant model (TT vs. CT+CC: OR = 0.742, 95% CI: 0.566-0.974, *P* = 0.031). However, the rs4821544 polymorphism was not correlated with RA susceptibility in male and female subjects, respectively. The results also indicated that rs729749 CT genotype frequency in RA patients was significantly lower than that in controls (CT vs. CC: OR = 0.758, 95% CI: 0.588-0.977, *P* = 0.033). Moreover, the rs729749 CT genotype frequency was significantly decreased in RA patients in males (CT vs. CC: OR = 0.487, 95% CI: 0.261-0.909, *P* = 0.024) and was not associated with RA susceptibility in females. No significant association between rs1883112 polymorphism and RA susceptibility was observed.

The genotype and allele frequencies of rs3794624 in *CYBA* were not significantly different among RA patients and controls (all *P* > 0.05), and no significant differences have existed in genotype and allele frequencies of *CYBA* rs4673 polymorphism (all *P* > 0.05). Similarly, *CYBA* rs3794624 and rs4673 polymorphisms were also not related to RA susceptibility in males and females, respectively.

### 3.2. Association of *NCF2*, *NCF4*, and *CYBA* Gene Polymorphisms with Clinical Features in RA Patients

Considering that RA was a heterogeneous disease, and different RF, anti-CCP antibody status might reflect disparate mechanisms of RA patients. We determined to evaluate whether *NCF2*, *NCF4*, and *CYBA* gene polymorphisms were associated with different serotypes of RA in a case-only study (Table 2). No significant differences have existed in *NCF2*, *NCF4*, and *CYBA* gene polymorphisms between RF-positive RA patients and RF-negative RA patients, as well as anti-CCP-positive RA patients and anti-CCP-negative RA patients, among the entire study population (all *P* > 0.05). Nonetheless, we found that the TT genotype and T allele frequencies of rs4821544 were significantly associated with anti-CCP in male RA patients (*P* = 0.031, *P* = 0.043).

### 3.3. Haplotype Analyses

In the present study, six main haplotypes (ATC, ATT, GCC, GCT, GTC, and GTT) for *NCF4* and three main haplotypes (AG, GA, and GG) for *CYBA* were detected by the SHEsis software. There was no significant difference regarding these haplotype frequencies between RA patients and normal controls (Tables 3 and 4).

## 4. Discussion

ROS was initially thought to be primarily involved in the chronic inflammation of autoimmune diseases such as SLE and RA. Then, the important regulatory functions of ROS were also observed in the immune system [8]. Additionally, some studies had attempted to explore whether *NOX* gene polymorphisms were related to multiple autoimmune diseases and suggested that *NOX* gene polymorphisms were associated with autoimmune diseases risk, as well as several specific clinical features [16, 22]. However, such studies were limited, especially in the Chinese population. To our knowledge, the present study was the first to analyze the relationship between polymorphisms of rs10911363 in *NCF2*; rs1883112, rs4821544, and rs729749 in *NCF4*; and rs3794624 and rs4673 in *CYBA* and RA susceptibility in a Chinese population. Since RA was more frequent in females than males, the disease mechanisms might be sex-dependent; hence, we stratified all subjects by sex and analyzed the association between all SNPs and RA susceptibility in males and females, respectively.

As a key component of the multiprotein NOX system, NCF2 was also called p67phox and encoded by the *NCF2* gene. The function of NCF2 was considered to regulate the transfer of electrons from NADPH to flavin, and phagocyte ROS production [23]. NCF2 was recruited to the cell membrane for combination with other components to form the active NOX system through microbial stimuli [24]. Moreover, mutations in the *NCF2* gene had been reported to affect the risk of a variety of diseases in previous studies [25]. Gateva et al. suggested that *NCF2* rs10911363 polymorphism was associated with SLE risk in the US and Sweden populations [26]. In another study, the *NCF2* rs789181 variant had been found to have a mild association with RA risk in men [16]. Here, we analyzed the possible relationship between rs10911363 polymorphism and RA susceptibility. However, the present result implied that rs10911363 might not be a contributing factor specific to RA susceptibility. Similarly, *NCF2* rs10911363 polymorphism exhibited no significant association with SLE risk in a Chinese population [20]. These interesting observations implied that *NCF2* gene rs10911363 polymorphism might not be involved in the pathogenesis of autoimmune diseases such as RA and SLE in a Chinese population. On the other hand, Yu et al. found that the rs10911363 G allele was positively correlated with several clinical characteristics and laboratory parameters in SLE patients and might influence the severity of this disease [20]. Therefore, the potential role of rs10911363 in RA development should be explored in future studies.

NCF4, known as a component of the NOX complex, could induce the NOX complex to phagosomal membranes through binding to phosphatidylinositol 3-phosphate (PtdIns3P) and had been proven to regulate the production of intracellular ROS [27, 28]. A recent animal experiment had explored the critical role of the NCF4-regulated intracellular ROS level in regulating chronic inflammation and autoimmunity, and the results found a mutation in the PtdIns3P-binding site of the regulatory NOX subunit NCF4/p40phox that could enhance autoimmune responses [29]. In another context, several genetic variants in the *NCF4* gene had been associated with autoimmune diseases including RA and Crohn's disease [16, 30, 31]. Roberts et al. revealed that *NCF4* rs4821544 polymorphism was significantly related to ileal Crohn's disease [30]. Similarly, our results demonstrated that rs4821544 CT genotype and C allele frequencies were significantly decreased in RA patients than controls. Olsson et al. found that rs729749 variant in *NCF4* was significantly associated with RA risk in men and observed a meaningful association for rs729749 in auto-antibody-negative disease, especially RF negative [16]. Consistent with this result, we found a significant association between rs729749 variation and RA risk in males, while our study also suggested that rs729749 polymorphism was significantly associated with RA susceptibility among the entire study population. The results from our study strengthened the hypothesis that the disease pathway affected by the rs729749 variation was specific to men. Moreover, our results also suggested a significant finding that the role of rs729749 variation in the pathogenesis of RA was closely related to race. In another study, the author found rs1883112 GG genotype frequency associated with a higher risk of diffuse large B-cell lymphoma [32]. However, there was no statistical association between rs1883112 variant and RA susceptibility in our study.

The present results suggested that TT genotype and T allele frequencies of rs4821544 were significantly lower in RA patients with anti-CCP-positive when compared to RA patients with anti-CCP-negative in male. This might help to develop a more appropriate therapeutic schedule for RA patients of different genders. However, it was worth noting that the sample of male RA patients with anti-CCP-negative in this study was small, and this result needed further verification. Our results found no association between rs729749 variants between RF and anti-CCP in RA patients. This was inconsistent with the results by Olsson et al. [16], possibly due to the different sample sizes and sources.

As a necessary subunit of NOX, p22phox was encoded by the *CYBA* gene and had an important role in regulating NOX activity. The other NOX subunits were expressed in thyroid and colon cells and paired with p22phox; therefore, p22phox was considered as an essential component in maintaining the function of NOX [33, 34]. A functional SNP (rs4673) had aroused great interest of many researchers, because it resulted in Tyr instead of His at residue 72 of p22phox, which has been suggested to significantly reduce basal and NAD(P)H-stimulated superoxide production [35]. Therefore, many studies had focused on the special role of *CYBA* gene variation in the development of multiple diseases. Lan et al. revealed that rs4673 polymorphism might lead to a high susceptibility of non-Hodgkin lymphoma [36]. Seibold et al. found some evidence for the association of *CYBA* rs3794624 polymorphism with postmenopausal breast cancer susceptibility [37]. Another meta-analysis showed a significant correlation between the rs4673 variant and T2DM susceptibility [38]. However, our data provide the first evidence that *CYBA* rs3794624 and rs4673 polymorphisms might not contribute to RA susceptibility in a Chinese population. Although rs3794624 and rs4673 polymorphisms showed no apparent link to RA risk, the accuracy of our results might be influenced by different genotyping, ethnicity, and environmental factors.

In conclusion, the present study demonstrated that *NCF4* rs4821544 and rs729749 polymorphisms were associated with RA susceptibility, and rs4821544 polymorphism was also related to anti-CCP in male RA patients. Furthermore, our findings strongly supported the viewpoint that the NOX system played an important role in the pathogenesis of RA. What is worth mentioning is that several limitations existed in the present study. Firstly, our study subjects might be insufficient, which resulted in the low power of this study. Secondly, this study did not detect the protein expression levels of these genes, and we were unable to further analyze the association between gene polymorphism and protein expression levels. Thirdly, special functional verification of relevant SNPs was missing; thus, the mechanism behind the results of genetic association was still unclear. The functional, replication studies with larger sample size, disparate races, and gene-environmental and gene-gene interaction are required.

## Figures and Tables

**Table 1:** The association between *NCF2*, *NCF4*, and *CYBA* gene polymorphisms and RA susceptibility among different groups (*n* (%)).

SNP	Analyze model	RA patients	Controls	*P* value	OR (95% CI)
All					
rs10911363	GG	125 (21.08)	144 (24.16)	0.371	0.863 (0.625, 1.192)
	GT	304 (51.26)	289 (48.49)	0.747	1.045 (0.798, 1.370)
	TT	164 (27.66)	163 (27.35)	Reference	
	G	554 (46.71)	577 (48.41)	0.408	1.070 (0.911, 1.257)
	T	632 (53.29)	615 (51.59)	Reference	
	TT	164 (27.66)	163 (27.35)	0.906	1.016 (0.787, 1.310)
	GT+GG	429 (72.34)	433 (72.65)	Reference	
	GG	125 (21.08)	144 (24.16)	0.204	0.838 (0.639, 1.101)
	GT+TT	468 (78.92)	452 (75.84)	Reference	
rs3794624	AA	14 (2.36)	15 (2.52)	0.929	0.967 (0.461, 2.027)
	GA	160 (26.98)	147 (24.66)	0.368	1.127 (0.868, 1.464)
	GG	419 (70.66)	434 (72.82)	Reference	
	A	188 (15.85)	177 (14.85)	0.498	1.080 (0.864, 1.350)
	G	998 (84.15)	1015 (85.15)	Reference	
	GG	419 (70.66)	434 (72.82)	0.408	0.899 (0.698, 1.157)
	GA+AA	174 (29.34)	162 (27.18)	Reference	
	AA	14 (2.36)	15 (2.52)	0.862	0.937 (0.448, 1.958)
	GA+GG	579 (97.64)	581 (97.48)	Reference	
rs4673	AA	1 (0.17)	5 (0.84)	0.140	0.198 (0.023, 1.698)
	GA	85 (14.33)	90 (15.10)	0.673	0.933 (0.677, 1.287)
	GG	507 (85.50)	501 (84.06)	Reference	
	A	87 (7.34)	100 (8.39)	0.340	0.864 (0.641, 1.166)
	G	1099 (92.66)	1092 (91.61)	Reference	
	GG	507 (85.50)	501 (84.06)	0.491	1.118 (0.814, 1.535)
	GA+AA	86 (14.50)	95 (15.94)	Reference	
	AA	1 (0.17)	5 (0.84)	0.142	0.200 (0.023, 1.714)
	GA+GG	592 (99.83)	591 (99.16)	Reference	
rs1883112	GG	57 (9.61)	56 (9.39)	0.972	1.007 (0.673, 1.508)
	GA	248 (41.82)	255 (42.79)	0.754	0.962 (0.757, 1.223)
	AA	288 (48.57)	285 (47.82)	Reference	
	G	362 (30.52)	367 (30.79)	0.888	0.988 (0.830, 1.176)
	A	824 (69.48)	825 (69.21)	Reference	
	AA	288 (48.57)	285 (47.82)	0.796	1.030 (0.821, 1.294)
	GA+GG	305 (52.43)	311 (52.18)	Reference	
	GG	57 (9.61)	56 (9.40)	0.899	1.025 (0.696, 1.511)
	GA+AA	536 (90.39)	540 (90.60)	Reference	
rs4821544	CC	4 (0.67)	7 (1.17)	0.323	0.536 (0.156, 1.845)
	CT	117 (19.73)	146 (24.50)	0.043	0.752 (0.571, 0.991)
	TT	472 (79.60)	443 (74.33)	Reference	
	C	125 (10.54)	160 (13.42)	0.031	1.316 (1.026, 1.688)
	T	1061 (89.46)	1032 (86.58)	Reference	
	TT	472 (79.60)	443 (74.33)	0.031	0.742 (0.566, 0.974)
	CT+CC	121 (20.40)	153 (25.67)	Reference	
	CC	4 (0.67)	7 (1.17)	0.374	0.571 (0.166, 1.962)
	CT+TT	589 (99.33)	589 (98.83)	Reference	
rs729749	TT	104 (17.54)	102 (17.11)	0.445	0.878 (0.628, 1.226)
	CT	266 (44.86)	302 (50.67)	0.033	0.758 (0.588, 0.977)
	CC	223 (37.61)	192 (32.21)	Reference	
	T	474 (39.97)	506 (42.45)	0.219	0.903 (0.767, 1.063)
	C	712 (60.03)	686 (57.55)	Reference	
	CC	223 (37.61)	192 (32.21)	0.051	1.268 (0.999, 1.611)
	CT+TT	370 (62.39)	404 (67.79)	Reference	
	TT	104 (17.54)	102 (17.11)	0.847	1.030 (0.763, 1.391)
	CT+CC	489 (82.46)	494 (82.89)	Reference	
Female					
rs10911363	GG	104 (21.14)	117 (23.45)	0.548	0.896 (0.625, 1.283)
	GT	259 (52.64)	252 (50.50)	0.818	1.036 (0.768, 1.397)
	TT	129 (26.22)	130 (26.05)	Reference	
	G	467 (47.46)	486 (48.70)	0.581	1.051 (0.881, 1.253)
	T	517 (52.54)	512 (51.30)	Reference	
	TT	129 (26.22)	130 (26.05)	0.952	0.991 (0.747, 1.316)
	GT+GG	363 (73.78)	369 (73.95)	Reference	
	GG	104 (21.14)	117 (23.45)	0.383	1.143 (0.847, 1.542)
	GT+TT	388 (78.86)	382 (76.55)	Reference	
rs3794624	AA	12 (2.44)	12 (2.40)	0.876	1.067 (0.473, 2.407)
	GA	137 (27.85)	121 (24.25)	0.194	1.208 (0.908, 1.607)
	GG	343 (69.72)	366 (73.35)	Reference	
	A	161 (16.36)	145 (14.53)	0.259	0.869 (0.681, 1.109)
	G	823 (83.64)	853 (85.47)	Reference	
	GG	343 (69.72)	366 (73.35)	0.205	1.195 (0.907, 1.576)
	GA+AA	149 (30.28)	133 (26.65)	Reference	
	AA	12 (2.44)	12 (2.40)	0.972	0.986 (0.438, 2.216)
	GA+GG	480 (97.56)	487 (97.60)	Reference	
rs4673	AA	1 (0.20)	5 (1.00)	0.140	0.198 (0.023, 1.703)
	GA	70 (14.23)	77 (15.43)	0.558	0.900 (0.634, 1.279)
	GG	421 (85.57)	417 (83.57)	Reference	
	A	72 (7.32)	87 (8.72)	0.252	1.210 (0.874, 1.675)
	G	912 (92.68)	911 (91.28)	Reference	
	GG	421 (85.57)	417 (83.57)	0.346	0.847 (0.600, 1.196)
	GA+AA	71 (14.43)	83 (16.63)	Reference	
	AA	1 (0.20)	5 (1.00)	0.144	4.970 (0.579, 42.692)
	GA+GG	491 (99.8)	494 (99.00)	Reference	
rs1883112	GG	41 (8.33)	47 (9.42)	0.630	0.894 (0.567, 1.409)
	GA	211 (42.89)	206 (41.28)	0.715	1.050 (0.808, 1.364)
	AA	240 (48.78)	246 (49.30)	Reference	
	G	293 (29.78)	300 (30.06)	0.890	1.014 (0.836, 1.229)
	A	691 (70.22)	698 (69.94)	Reference	
	AA	240 (48.78)	246 (49.30)	0.870	1.021 (0.796, 1.310)
	GA+GG	252 (51.22)	253 (50.70)	Reference	
	GG	41 (8.33)	47 (9.42)	0.548	1.144 (0.738, 1.774)
	GA+AA	451 (91.67)	452 (90.58)	Reference	
rs4821544	CC	4 (0.81)	6 (1.20)	0.494	0.641 (0.180, 2.290)
	CT	95 (19.31)	115 (23.05)	0.141	0.795 (0.585, 1.079)
	TT	393 (79.88)	378 (75.75)	Reference	
	C	103 (10.47)	127 (12.73)	0.117	1.247 (0.946, 1.644)
	T	881 (89.53)	871 (87.27)	Reference	
	TT	393 (79.88)	378 (75.75)	0.118	0.787 (0.583, 1.063)
	CT+CC	99 (20.12)	121 (24.25)	Reference	
	CC	4 (0.81)	6 (1.20)	0.540	1.485 (0.416, 5.294)
	CT+TT	488 (99.19)	493 (98.80)	Reference	
rs729749	TT	84 (17.07)	87 (17.43)	0.469	0.873 (0.604, 1.261)
	CT	231 (46.95)	252 (50.50)	0.186	0.829 (0.627, 1.095)
	CC	177 (35.98)	160 (32.06)	Reference	
	T	399 (40.55)	426 (42.69)	0.335	1.092 (0.913, 1.306)
	C	585 (59.45)	572 (57.31)	Reference	
	CC	177 (35.98)	160 (32.06)	0.194	0.840 (0.646, 1.093)
	CT+TT	315 (64.02)	339 (67.94)	Reference	
	TT	84 (17.07)	87 (17.43)	0.880	1.026 (0.738, 1.426)
	CT+CC	408 (82.93)	412 (82.57)	Reference	
Male					
rs10911363	GG	21 (20.79)	27 (27.84)	0.413	0.733 (0.349, 1.541)
	GT	45 (44.55)	37 (38.14)	0.677	1.147 (0.602, 2.184)
	TT	35 (34.65)	33 (34.02)	Reference	
	G	87 (43.07)	91 (46.91)	0.443	1.168 (0.786, 1.736)
	T	115 (56.93)	103 (53.09)	Reference	
	TT	35 (34.65)	33 (34.02)	0.925	0.972 (0.541, 1.749)
	GT+GG	66 (65.35)	64 (65.98)	Reference	
	GG	21 (20.79)	27 (27.84)	0.249	1.469 (0.764, 2.827)
	GT+TT	80 (79.21)	70 (72.16)	Reference	
rs3794624	AA	2 (1.98)	3 (3.09)	0.578	0.596 (0.097, 3.677)
	GA	23 (22.77)	26 (26.8)	0.480	0.791 (0.413, 1.515)
	GG	76 (75.25)	68 (70.1)	Reference	
	A	27 (13.37)	32 (16.49)	0.383	1.280 (0.735, 2.230)
	G	175 (86.63)	162 (83.51)	Reference	
	GG	76 (75.25)	68 (70.1)	0.417	0.771 (0.412, 1.444)
	GA+AA	25 (24.75)	29 (29.9)	Reference	
	AA	2 (1.98)	3 (3.09)	0.621	1.580 (0.258, 9.666)
	GA+GG	99 (98.02)	94 (96.91)	Reference	
rs4673	AA	0	0	—	—
	GA	15 (14.85)	13 (13.40)	0.770	1.127 (0.506, 2.511)
	GG	86 (85.15)	84 (86.60)	Reference	
	A	15 (7.43)	13 (6.70)	0.779	0.895 (0.414, 1.934)
	G	187 (92.57)	181 (93.30)	Reference	
rs1883112	GG	16 (15.84)	9 (9.28)	0.433	1.444 (0.576, 3.623)
	GA	37 (36.63)	49 (50.52)	0.111	0.614 (0.336, 1.119)
	AA	48 (47.52)	39 (40.21)	Reference	
	G	69 (34.16)	67 (34.54)	0.937	1.017 (0.672, 1.540)
	A	133 (65.84)	127 (65.46)	Reference	
	AA	48 (47.52)	39 (40.21)	0.300	0.742 (0.423, 1.304)
	GA+GG	53 (52.48)	58 (59.79)	Reference	
	GG	16 (15.84)	9 (9.28)	0.169	0.543 (0.228, 1.296)
	GA+AA	85 (84.16)	88 (90.72)	Reference	
rs4821544	CC	0	1 (1.03)	1.000	—
	CT	22 (21.78)	31 (31.96)	0.098	0.584 (0.309, 1.104)
	TT	79 (78.22)	65 (67.01)	Reference	
	C	22 (10.89)	33 (17.01)	0.081	1.677 (0.939, 2.995)
	T	180 (89.11)	161 (82.99)	Reference	
	TT	79 (78.22)	65 (67.01)	0.078	0.566 (0.300, 1.067)
	CT+CC	22 (21.78)	32 (32.99)	Reference	
	CC	0	1 (1.03)	1.000	—
	CT+TT	101 (100.00)	96 (98.97)	Reference	
rs729749	TT	20 (19.8)	15 (15.46)	0.855	0.928 (0.414, 2.079)
	CT	35 (34.65)	50 (51.55)	0.024	0.487 (0.261, 0.909)
	CC	46 (45.54)	32 (32.99)	Reference	
	T	75 (37.13)	80 (41.24)	0.403	1.188 (0.793, 1.780)
	C	127 (62.87)	114 (58.76)	Reference	
	CC	46 (45.54)	32 (32.99)	0.072	0.589 (0.331, 1.041)
	CT+TT	55 (54.46)	65 (67.01)	Reference	
	TT	20 (19.80)	15 (15.46)	0.425	0.741 (0.355, 1.547)
	CT+CC	81 (80.20)	82 (84.54)	Reference	

**Table 2:** Association of clinical characteristics with genotype and allele frequencies in *NCF2*, *NCF4*, and *CYBA* genes among different groups (*n* (%)).

SNP	Allele	Clinical features	Group	Genotypes *n* (%)			*P* value	Alleles *n* (%)		*P* value
	(M/m)			MM	Mm	mm		M	m	
All										
rs10911363	T/G	Anti-CCP	Positive	128 (26.28)	255 (52.36)	104 (21.35)	0.110	511 (52.46)	463 (47.54)	0.206
			Negative	28 (37.33)	31 (41.33)	16 (21.33)		87 (58.00)	63 (42.00)	
		RF	Positive	126 (26.30)	256 (53.44)	97 (20.25)	0.122	508 (53.03)	450 (46.97)	0.806
			Negative	30 (30.93)	41 (42.27)	26 (26.80)		101 (52.06)	93 (47.94)	
rs3794624	G/A	Anti-CCP	Positive	346 (71.05)	132 (27.10)	9 (1.85)	0.172	824 (84.60)	150 (13.40)	0.416
			Negative	52 (69.33)	19 (25.33)	4 (5.33)		123 (82.00)	27 (18.00)	
		RF	Positive	340 (70.98)	128 (26.72)	11 (2.30)	0.682	808 (84.34)	150 (15.66)	0.946
			Negative	68 (70.10)	28 (28.87)	1 (1.03)		164 (84.54)	30 (15.46)	
rs4673	G/A	Anti-CCP	Positive	421 (86.45)	66 (13.55)	0	0.238	908 (93.22)	66 (6.78)	0.257
			Negative	61 (81.33)	14 (28.67)	0		136 (90.67)	14 (9.33)	
		RF	Positive	409 (85.39)	69 (14.41)	1 (0.21)	0.904	887 (92.59)	71 (7.41)	0.925
			Negative	83 (85.57)	14 (14.43)	0		180 (92.78)	14 (7.22)	
rs1883112	A/G	Anti-CCP	Positive	241 (49.49)	201 (41.27)	45 (9.24)	0.880	683 (70.12)	291 (29.88)	0.976
			Negative	36 (48.00)	33 (44.00)	6 (8.00)		105 (70.00)	45 (30.00)	
		RF	Positive	238 (49.69)	192 (40.08)	49 (10.23)	0.076	668 (69.73)	290 (30.27)	0.697
			Negative	45 (46.39)	48 (49.48)	4 (4.12)		138 (71.13)	56 (28.87)	
rs4821544	T/C	Anti-CCP	Positive	384 (78.85)	99 (20.33)	4 (0.82)	0.598	867 (89.01)	107 (10.99)	0.392
			Negative	62 (82.67)	13 (17.33)	0		137 (91.33)	13 (8.67)	
		RF	Positive	378 (78.91)	97 (20.25)	4 (0.84)	0.609	853 (89.04)	105 (10.96)	0.489
			Negative	79 (81.44)	18 (18.56)	0		176 (90.72)	18 (9.28)	
rs729749	C/T	Anti-CCP	Positive	188 (38.60)	215 (44.15)	84 (17.25)	0.820	591 (60.68)	383 (39.32)	0.754
			Negative	29 (38.67)	31 (41.33)	15 (20.00)		89 (59.33)	61 (40.67)	
		RF	Positive	183 (38.20)	215 (44.89)	81 (16.91)	0.676	581 (60.65)	377 (39.35)	0.533
			Negative	36 (37.11)	41 (42.27)	20 (20.62)		113 (58.25)	81 (41.75)	
Female										
rs10911363	T/G	Anti-CCP	Positive	86 (21.18)	218 (53.69)	102 (25.12)	0.162	390 (48.03)	422 (51.97)	0.265
			Negative	13 (21.31)	26 (42.62)	22 (36.07)		52 (42.62)	70 (57.38)	
		RF	Positive	98 (24.69)	220 (55.42)	79 (19.9)	0.074	416 (52.39)	378 (47.61)	0.680
			Negative	24 (29.63)	34 (41.98)	23 (28.40)		82 (50.62)	80 (49.38)	
rs3794624	G/A	Anti-CCP	Positive	283 (69.70)	115 (28.33)	8 (1.97)	0.360	681 (83.87)	131 (16.13)	0.597
			Negative	42 (68.85)	16 (26.23)	3 (4.92)		100 (81.97)	22 (18.03)	
		RF	Positive	277 (69.77)	110 (27.71)	10 (2.52)	0.750	664 (83.63)	130 (16.37)	0.919
			Negative	56 (69.14)	24 (29.63)	1 (1.23)		136 (83.95)	26 (16.05)	
rs4673	G/A	Anti-CCP	Positive	352 (86.70)	54 (13.30)	0	0.096	109 (89.34)	13 (10.66)	0.110
			Negative	48 (78.69)	13 (21.31)	0		758 (93.35)	54 (6.65)	
		RF	Positive	341 (85.89)	55 (13.85)	1 (0.25)	0.660	737 (92.82)	57 (7.18)	0.517
			Negative	67 (82.72)	14 (17.28)	0		148 (91.36)	14 (8.64)	
rs1883112	A/G	Anti-CCP	Positive	202 (49.75)	171 (42.12)	33 (8.13)	0.844	575 (70.81)	237 (29.19)	0.658
			Negative	28 (45.90)	28 (45.90)	5 (8.20)		84 (68.85)	38 (31.15)	
		RF	Positive	201 (50.63)	162 (40.81)	34 (8.56)	0.151	564 (71.03)	230 (28.97)	0.629
			Negative	35 (43.21)	42 (51.85)	4 (4.94)		112 (69.14)	50 (30.86)	
rs4821544	T/C	Anti-CCP	Positive	324 (79.80)	78 (19.21)	4 (0.99)	0.694	726 (89.41)	86 (10.59)	0.983
			Negative	48 (78.69)	13 (21.31)	0		109 (89.34)	13 (10.66)	
		RF	Positive	317 (79.85)	76 (19.14)	4 (1.01)	0.625	710 (89.42)	84 (10.58)	0.974
			Negative	64 (79.01)	17 (20.99)	0		145 (89.51)	17 (10.49)	
rs729749	C/T	Anti-CCP	Positive	150 (36.95)	187 (46.06)	69 (17.00)	0.978	487 (59.98)	325 (40.02)	0.840
			Negative	22 (36.07)	28 (45.90)	11 (18.03)		72 (59.02)	50 (40.98)	
		RF	Positive	148 (37.28)	184 (46.35)	65 (16.37)	0.513	480 (60.45)	314 (39.55)	0.247
			Negative	26 (32.10)	38 (46.91)	17 (20.99)		90 (55.56)	72 (44.44)	
Male										
rs10911363	T/G	Anti-CCP	Positive	26 (32.10)	37 (45.68)	18 (22.22)	0.711	89 (54.94)	73 (45.06)	0.570
			Negative	6 (42.86)	5 (35.71)	3 (21.43)		17 (60.71)	11 (39.29)	
		RF	Positive	28 (34.15)	36 (43.90)	18 (21.95)	0.948	92 (56.10)	72 (43.90)	0.732
			Negative	6 (37.50)	7 (43.75)	3 (18.75)		19 (59.38)	13 (40.63)	
rs3794624	G/A	Anti-CCP	Positive	63 (77.78)	17 (20.99)	1 (1.23)	0.360	143 (88.27)	19 (11.73)	0.367
			Negative	10 (71.43)	3 (21.43)	1 (7.14)		23 (82.14)	5 (17.86)	
		RF	Positive	63 (76.83)	18 (21.95)	1 (1.22)	0.880	144 (87.80)	20 (12.20)	0.962
			Negative	12 (75.00)	4 (25.00)	0		28 (87.50)	4 (12.50)	
rs4673	G/A	Anti-CCP	Positive	69 (85.19)	12 (14.81)	0	0.441	150 (92.59)	12 (7.41)	0.458
			Negative	13 (92.86)	1 (7.14)	0		27 (96.43)	1 (3.57)	
		RF	Positive	68 (82.93)	14 (17.07)	0	0.074	150 (91.46)	14 (8.54)	0.086
			Negative	16 (100.00)	0	0		32 (100.00)	0	
rs1883112	A/G	Anti-CCP	Positive	39 (48.15)	30 (37.04)	12 (14.81)	0.700	108 (66.67)	54 (33.33)	0.383
			Negative	8 (57.14)	5 (35.71)	1 (7.14)		21 (75.00)	7 (25.00)	
		RF	Positive	37 (45.12)	30 (36.59)	15 (18.29)	0.152	104 (63.41)	60 (36.59)	0.051
			Negative	10 (62.50)	6 (37.50)	0		26 (81.25)	6 (18.75)	
rs4821544	T/C	Anti-CCP	Positive	60 (74.07)	21 (25.93)	0	0.031	141 (87.04)	21 (12.96)	0.043
			Negative	14 (100.00)	0	0		28 (100.00)	0	
		RF	Positive	61 (74.39)	21 (25.61)	0	0.090	143 (87.20)	21 (12.80)	0.113
			Negative	15 (93.75)	1 (6.25)	0		31 (96.88)	1 (3.13)	
rs729749	C/T	Anti-CCP	Positive	38 (46.91)	28 (34.57)	15 (18.52)	0.533	104 (64.20)	58 (35.80)	0.723
			Negative	7 (50.00)	3 (21.43)	4 (28.57)		17 (60.71)	11 (39.29)	
		RF	Positive	35 (42.68)	31 (37.80)	16 (19.51)	0.279	101 (61.59)	63 (38.41)	0.269
			Negative	10 (62.50)	3 (18.75)	3 (18.75)		23 (71.88)	9 (28.13)	

M: major alleles; m: minor alleles.

**Table 3:** Haplotype analysis of three SNPs in the *NCF4* gene in RA patients and controls (*n* (%)).

Haplotype	RA patients	Controls	*P* value	OR (95% CI)
rs1883112-rs4821544-rs729749				
ATC	525.82 (44.3)	502.44 (42.2)	0.282	1.093 (0.929, 1.286)
ATT	298.18 (25.1)	322.56 (27.1)	0.286	0.905 (0.754, 1.087)
GCC	97.99 (8.3)	118.49 (9.9)	0.155	0.816 (0.616, 1.080)
GCT	27.01 (2.3)	41.51 (3.5)	0.079	0.646 (0.395, 1.056)
GTC	88.19 (7.4)	65.06 (5.5)	0.050	1.391 (0.999, 1.937)
GTT	148.81 (12.5)	141.94 (11.9)	0.634	1.061 (0.830, 1.357)

Frequency < 0.03 in both controls and RA patients has been dropped.

**Table 4:** Haplotype analysis of two SNPs in the *CYBA* gene in RA patients and controls (*n* (%)).

Haplotype	RA patients	Controls	*P* value	OR (95% CI)
rs3794624-rs4673				
AG	179.43 (15.1)	173.64 (14.6)	0.667	1.051 (0.838, 1.318)
GA	78.43 (6.6)	96.64 (8.1)	0.172	0.806 (0.592, 1.099)
GG	919.57 (77.5)	918.36 (77.0)	0.625	1.049 (0.865, 1.274)

Frequency < 0.03 in both controls and RA patients has been dropped.

## Data Availability

The data that support the findings of this study are available from the corresponding authors upon reasonable request.
